# Some American Hospitals

**Published:** 1892-02-20

**Authors:** 


### Some American Hospitals.

By Our Special Commissioner.

### VIII.—THE UNIVERSITY OF PENNSYLVANIA HOSPITAL.

This institution was reorganised recently, and placed under the direction of Dr. J. S. Billings. It is unnecessary to say that as a result of Dr. Billing's management the whole hospital has undergone a complete renovation, and it may be favourably compared with any similar institution in Philadelphia. The drainage, plumbing, interior decoration, floors, operation theatre, laundry, lavatories, and in fact, the whole of the buildings have been thoroughly overhauled, re-constructed, and modernised. A new mortuary, with a mortuary chapel, has been erected at the back of the hospital. The accommodation for paying patients has been greatly increased, and it is believed that the income derived from them will ultimately be sufficient to enable the trustees to free themselves from debt by paying off the mortgages. With the view of strengthening the financial administration, the Board of Managers have applied to the legislature for State aid to the extent of seventy thousand dollars. The wards are pleasant in appearance, excellently kept, and thoroughly comfortable. The staff seem to take a genuine interest in their work. The temperature of the hospital is so adjusted as to make it much more pleasant as a residence than many of those we have inspected during our visit to the United States. The hospital is inadequately supported at the present time, as the debt for current supplies amounts to 33,531 dollars. The hospital has accommodation for about 120 patients, the average number under treatment last year having been about 100. The average stay and residence of each patient was 26 days. Total expenditure about 60,000 dollars. The average cost of maintenance for each patient per diem is 7s., or $1.74 of the whole number of in-patients admitted, viz., 1,181, 656 or more than one-half were pay patients. These patients appear to have paid altogether 20,000 dollars for their accommodation, or about 30 dollars each. The actual average expense charged to a patient per diem was 8s., or $1.95.

### Nursing Department.

This hospital has a nursing home, and the whole department is in charge of the Superintendent, Miss Davis. The school was organised in 1886, and has now become an important feature of the hospital. There are thirty nurses in course of training. Instruction is given by lectures, demonstrations, and practical teaching, the whole of the arrangements being in the immediate charge of the Assistant Superintendent. The Committee give a medal each year to the nurse who graduates with the highest honours. It is called the Nightingale Medal, and has so far been awarded to Mary Clymer, Mary Fisher, and Caroline Slees. The home was erected in 1886, and presented to the hospital as a memorial to Mrs. Richard D. Wood. The special feature of the school, and one which attracts educated women here for training, is the large number of private patients in the hospital, which affords an opportunity for special instruction to the nurses in the advanced classes, who are also given much individual responsibility in the private wards. These advantages prove of the utmost value to the nurses when they first engage in private work after they have graduated.

